# Measuring Success of Patients’ Continuous Use of Mobile Health Services for Self-management of Chronic Conditions: Model Development and Validation

**DOI:** 10.2196/26670

**Published:** 2021-07-13

**Authors:** Ting Song, Ning Deng, Tingru Cui, Siyu Qian, Fang Liu, Yingping Guan, Ping Yu

**Affiliations:** 1 Centre for Digital Transformation, School of Computing and Information Technology Faculty of Engineering and Information Sciences University of Wollongong Wollongong Australia; 2 Illawarra Health and Medical Research Institute University of Wollongong Wollongong Australia; 3 The Ministry of Education Key Laboratory of Biomedical Engineering, College of Biomedical Engineering and Instrument Science Zhejiang University Huangzhou China; 4 School of Computing and Information Systems Faculty of Engineering and Information Technology University of Melbourne Melbourne Australia; 5 Drug and Alcohol Service Illawarra Shoalhaven Local Health District Wollongong Australia; 6 Department of Health Examination General Hospital of Ningxia Medical University Yinchuan China

**Keywords:** mobile health, service, smartphone, mobile application, continuous use, high blood pressure, chronic disease, PLS

## Abstract

**Background:**

Mobile health services are gradually being introduced to support patients’ self-management of chronic conditions. The success of these services is contingent upon patients’ continuous use of them.

**Objective:**

This study aims to develop a model to measure the success of patients’ continuous use of mobile health services for the self-management of chronic conditions.

**Methods:**

The proposed model was derived from the information systems continuance model and the information systems success model. This model contains 7 theoretical constructs: information quality, system quality, service quality, perceived usefulness, user satisfaction, perceived health status, and continuous use intention. A web-based questionnaire survey instrument was developed to test the model. The survey was conducted to collect data from 129 patients who used a mobile health app for hypertension management from 2017 to 2019. The questionnaire items were derived from validated instruments and were measured using a 5-point Likert scale. The partial least squares modelling method was used to test the theoretical model.

**Results:**

The model accounted for 58.5% of the variance in perceived usefulness (R^2^=0.585), 52.3% of the variance in user satisfaction (R^2^=0.523), and 41.4% of the variance in patients’ intention to make continuous use of mobile health services (R^2^=0.414). The continuous use intention was significantly influenced by their perceived health status (β=.195, *P*=.03), perceived usefulness (β=.307, *P*=.004), and user satisfaction (β=.254, *P*=.04) with the mobile health service. Information quality (β=.235, *P*=.005), system quality (β=.192, *P*=.02), and service quality (β=.494, *P*<.001) had a significantly positive influence on perceived usefulness but not on user satisfaction. Perceived usefulness had a significantly positive influence on user satisfaction (β=.664, *P*<.001). In a result opposite to the original hypothesis, perceived health status did not negatively influence patients’ intention to continue using the mobile health service but showed a significantly positive correlation.

**Conclusions:**

This study developed a theoretical model to predict and explain patients’ continuous use of mobile health services for self-management of chronic conditions and empirically tested the model. Perceived usefulness, user satisfaction, and health status contributed to patients’ intention to make continuous use of mobile health services for self-managing their chronic conditions.

## Introduction

### Background

Mobile health (mHealth) services have been increasingly introduced to support patients in their self-management of chronic conditions over the last decade [1,2]. mHealth overcomes the traditional barriers of time, distance, and cost by providing patients with access to health information, assessment, and assistance anytime and anywhere [3,4]. As one of the most used mHealth services, mHealth apps are widely used to record and evaluate patients’ vital signs and self-management behaviors such as medication, exercise, and diet; access health information; remind self-management behaviors; and communicate with health care providers [1,2,5]. With increasing awareness and ability, patients are motivated to self-manage their behavior, that is, adhere to treatment, thereby controlling their chronic conditions and maintaining the quality of life. When used over a long period of time, these apps are likely to result in positive outcomes such as obvious changes in health behaviors [6-8]. Despite their potential benefits, mHealth apps are rarely used. Perez [9] found that 25% of mHealth apps were used only once after installation, and most users stopped using these apps after 4 periods of interaction with the apps. A national survey conducted in the United States showed that 45.7% (427/934) of the participants who downloaded certain mHealth apps no longer used these apps [10]. This is contrary to the original intention of introducing these apps to help the management of long-term diseases because short-term use is not sufficient to achieve the expected benefits [11,12]. Therefore, it is essential to understand the factors that affect patients’ continuous use of mHealth apps.

Prior studies on patients’ use of mHealth apps have focused on patient acceptance and initial use of these apps [13-22], whereas only a few studies have focused on the continuous use of mHealth services [23-26]. Some of these continuous use studies only examined mHealth services for general health care, such as appointments and health consultations instead of self-management of chronic conditions [23,24,26]. Cho [26] developed and tested a model to explain the mechanism that determines the continuous use intention of mHealth services. He found that continuous use intention is influenced by confirmation of the primary expectation of mHealth apps but he did not specify the content of the expectation. Lee et al [25] found that patients’ intention to continuously use an mHealth service was closely associated with their regular use of self-monitoring functions; however, they did not further explore the relationship between these 2 constructs.

To date, there has been little theoretical research on the factors influencing patients’ continuous use of mHealth apps to support self-management of chronic conditions. To fill this gap, this research aims to (1) identify the influencing factors and develop a predictive model to explain their relationships with patients’ continuous use of mHealth apps for self-management of chronic conditions, (2) develop and validate a questionnaire survey instrument that empirically tests and theorizes the model, and (3) examine the associations among the variables and their relative impact on the continuous use of an mHealth app. The theoretical model was tested in the context of hypertension self-management.

### Theoretical Foundation

The constructs of the proposed model are drawn from the information systems (IS) continuance model [27] and IS success model [28]. Inspired by Oliver’s Expectation Confirmation Theory [29], Bhattacherjee [27] proposed that information system users’ continuous use intention is similar to their repurchase decision-making. They compared the benefits acquired from using an information system product or service with the prior expectation to decide the level of satisfaction with it. The comparison result, that is, confirmation, became their reference for continuous use. This theory is known as the IS continuance model. In this model, perceived usefulness and user satisfaction are considered crucial factors influencing continuous use intention and are associated with confirmation, thus being deemed intermediate variables. However, the content of the confirmation is not clearly defined in this model, which makes it difficult to assess the factors that determine the continuous use intention.

In their famous IS success model, Delone and McLean [28] proposed that 3 factors, namely, information quality, system quality, and service quality determine use intention and satisfaction, and use intention and satisfaction predict information system success. This theory has been widely adopted in studies that evaluate information system success [30,31]. According to a published mobile technology acceptance model, usefulness predicts use intention [13]. Therefore, we posit that information quality, system quality, and service quality may affect perceived usefulness and user satisfaction of mHealth services for patient self-management of chronic conditions.

### Research Hypotheses

#### Information Quality, System Quality, Service Quality, and Perceived Usefulness

Information quality refers to the quality of content that the mobile service provides. Its attributes include relevance, timeliness of update, and ease of understanding [28,32]. Perceived usefulness refers to users’ ex-post expectations and beliefs about the effectiveness and benefits of using an mHealth app from their experience [27]. Information quality is one of the critical determinants for information system success because the acquisition of information is the main purpose for users to use an information system. Alsabawy et al [33] found that low-quality information provided by an e-learning system may mislead users and consequently change their perception of its usefulness. System quality refers to the overall performance of an mHealth app as perceived by the users [28,32]. It measures the technical success of an mHealth service. As an information system is the carrier of information, its quality is the prerequisite for ensuring that users can easily obtain the information they need. For example, if the functions of an information system are too complex and difficult to use, users may not invest time and energy to learn and use the system, which may weaken users’ perception of the usefulness of the system [30]. Service quality refers to the support that an mHealth app user can receive from the support personnel and technical team who administer the system [28,32]. The attributes include dependability, availability, and empathy of the support staff. Dou et al [13] found that a good doctor-patient relationship was one of the prerequisites for patients to think that an mHealth service was useful to them. For example, they thought the mHealth service was useful only if the patients knew the health care provider could help them solve problems through the app. Wu [31] also found that high-quality service can increase users’ perceived usefulness of a web-based health care community. Watts et al [34] suggested that assessing the relationships between these information system factors needs to consider the context of information system use. Therefore, the following hypotheses were posited:

Hypothesis 1(a): *Information quality is positively associated with patients’ perceived usefulness of mHealth services.*

Hypothesis 1(b): *System quality is positively associated with patients’ perceived usefulness of mHealth services.*

Hypothesis 1(c): *Service quality is positively associated with patients’ perceived usefulness of mHealth services.*

#### Information Quality, System Quality, Service Quality, and User Satisfaction

User satisfaction refers to a user’s emotional or psychological state about using a system [28]. Delone and McLean [28] posited that system quality, information quality, and service quality predict user satisfaction. For example, patients are dissatisfied with irrelevant information in the app, such as overwhelming advertisements [24]. Users prefer a clean and simple interface and easy-to-understand navigation menu. Zheng et al [32] reported that if the service provided by an information system does not provide the expected reliability or consistency, satisfaction with the information system will decrease. Consequently, the following hypotheses were posited:

Hypothesis 2(a): *Information quality is positively associated with user satisfaction of mHealth services.*

Hypothesis 2(b): *System quality is positively associated with user satisfaction of mHealth services.*

Hypothesis 2(c): *Service quality is positively associated with user satisfaction of mHealth services.*

#### Perceived Usefulness, User Satisfaction, Perceived Health Status, and Continuous Use Intention

Bhattacherjee [27] suggested that perceived usefulness predicts user satisfaction due to a likely positive emotional response derived from improvement in work efficiency and job performance by using the information system [35]. With this understanding, Kim and Lee [36] focused on investigating the usefulness of their robot services to improve user satisfaction. Likewise, if patients believe that using a mHealth app can help them control hypertension, they should also be satisfied with the app. Therefore, the following hypothesis was posited:

Hypothesis 3: *Perceived usefulness of mHealth services is positively associated with user satisfaction with such services.*

Perceived health status refers to individuals’ assessments of their health conditions [37]. It reflects the physiological, behavioral, social, and psychological conditions that a person experiences, which can be difficult to capture by other objective indicators [38]. Song et al [8] found that many patients reduced usage frequency or even stopped using the mHealth service once their blood pressure was under control. However, they would resume use once their blood pressure increased again.

Bhattacherjee [27] showed that perceived usefulness and user satisfaction will positively predict continuous use intention. Vaghefi and Tulu [24] interviewed 17 people who used different apps to support their lifestyle changes, that is, promoting physical activity and mindfulness. The comparison of user perceptions 2 weeks before and after app usage indicated that user experience and perceived health goals are 2 factors influencing these people’s continuous use intention. Therefore, the following hypotheses were posited:

Hypothesis 4(a): *Perceived health status is negatively* *associated with continuous use intention of mHealth services.*

Hypothesis 4(b): *Perceived usefulness is positively associated with continuous use intention of mHealth services.*

Hypothesis 4(c): *User satisfaction is positively associated with continuous use intention of mHealth services.*

### The Proposed Theoretical Model

**Figure 1:**
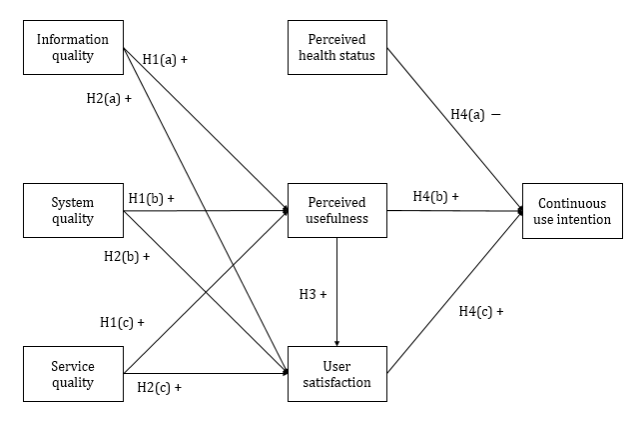
The proposed research model. "H" refers to the hypotheses in this study. +: positive association; –: negative association.

The proposed continuous use model of mHealth services for self-management of chronic conditions consists of 7 variables (Figure 1). Four of them are independent variables: information quality, system quality, service quality, and perceived health status. Continuous use intention is the dependent variable. The remaining 2 are mediators: perceived usefulness and user satisfaction.

## Methods

### Ethics Approval

This study was approved by the Human Research Ethics Committee of the General Hospital of Ningxia Medical University, China (Registration number: 2018-325).

### Study Setting and mHealth Service

To improve patient hypertension management and population health, an international tripartite collaborative research program was piloted. The mHealth service entitled “Blood Pressure Assistant” was developed by the Biomedical Informatics Laboratory at Zhejiang University, China, to assist outpatients in self-managing hypertension. The mHealth service included an app for the patients to use and a web-based portal for health care providers, including clinicians and certified health managers, to communicate with the patients. The functional modules of the app were (1) digital forms to record vital signs, that is, blood pressure, heart rate, weight, and self-management behaviors, that is, medication, exercise, and diet; (2) health education materials; (3) reminders for self-management; (4) reports on daily/monthly statistical trend of vital signs and self-management performance; and (5) feedback about the blood pressure level, being normal or not. The functional modules of the web-based portal included (1) statistical results and visualization charts of data recorded by patients; (2) alerts on abnormal situations; and (3) patient classification and follow-up tracking reminders. The mHealth service was implemented in the Department of Cardiology, General Hospital of Ningxia Medical University, China, in November 2015. The effectiveness of this service was evaluated in a clinical trial (Registration number: ChiCTR1900026437). The trial participants were outpatients with hypertension of the hospital and were provided with a 1-hour face-to-face training before using the app. The training included the significance and methods of hypertension self-management, the way to install and use the app, and tips to solve common usage problems.

### Questionnaire Survey Development and Data Collection

A self-administered questionnaire was developed in 2017 to collect data to measure the 7 latent variables by using a 5-point Likert scale (Table 1) and to test the relationships among them. The questionnaire consisted of 17 questions, and all were adopted from previously validated studies and modified to fit our study context. Although each latent construct is best measured by at least 3 items [39], in balancing rigor with avoiding survey fatigue for satisfactory response rates [40], we only used 2 items to measure 5 constructs in reference to the recommendation of previous literature [13,26,30]. Each question was anchored between 1 (strongly disagree) and 5 (strongly agree). The measurement items were translated into Chinese by one researcher and then discussed and validated by a panel of 9 bilingual experts, that is, 4 clinicians, 3 medical informatics experts, 1 certified health manager, and 1 information system expert. One researcher then back translated the instrument into English to confirm the accuracy and quality of the Chinese translation.

**Table 1:** Questionnaire constructs and their measurement items scored using a 5-point Likert scale.

Construct, item code			Item description [citations of validated studies]		Score, mean (SD)^a^
**Information quality (IQ)**					
	IQ1	Information provided by the BP^b^ Assistant is relevant for my needs [28,29,32].		4.310 (0.745)	
	IQ2	Information provided by the BP Assistant is sufficient for my needs [28,32].		4.318 (0.757)	
	IQ3	Information provided by the BP Assistant has been updated in a timely manner [28,32].		3.953 (0.879)	
**System quality (SysQ)**					
	SysQ1	The BP Assistant is easy to learn [29].		4.519 (0.572)	
	SysQ2	The BP Assistant is easy to use [28,29].		4.481 (0.624)	
**Service quality (SerQ)**					
	SerQ1	The health manager can resolve problems that I encountered when using the BP Assistant [28].		4.271 (0.745)	
	SerQ2	The health manager gives me individual guidance in the follow-up phone calls [28,32].		4.085 (0.778)	
**Perceived usefulness (PU)**					
	PU1	The BP Assistant is of benefit to me [26].		4.202 (0.730)	
	PU2	Overall, using the BP Assistant is advantageous to my hypertension management [27].		4.380 (0.587)	
**User satisfaction (US)**					
	US1	I am content with my use of BP Assistant [27,41,42].		4.504 (0.599)	
	US2	I am satisfied with my use of BP Assistant [27,29,41,42].		4.488 (0.544)	
**Perceived health status (PHS)**					
	PHS1	Currently, my BP is back to normal [8,43].		4.101 (0.796)	
	PHS2	Currently, my BP is under control [8,43,44].		4.031 (0.787)	
	PHS3	Currently, I don’t worry about my health [13].		4.225 (0.707)	
**Continuous use intention (CUI)**					
	CUI1	I intend to continue using the BP Assistant rather than discontinue its use [27,42].		4.519 (0.558)	
	CUI2	If I could, I would like to discontinue my use of the BP Assistant (reverse coded) [27].		4.116 (0.850)	

^a^Scored on a 5-point Likert scale. Each question was anchored between 1 (strongly disagree) and 5 (strongly agree).

^b^BP: blood pressure.

The questionnaire survey was then built into the mHealth app as a special functional module for the trial participants to complete. A participant information sheet was placed at the beginning of the questionnaire to inform them about the purpose of the survey, the voluntary nature of completing it, and the confidentiality and anonymity of their responses for any derived research publication. The participants could click a checkbox to express their consent. Some participants returned the completed questionnaire without clicking this checkbox, and this was treated as implied consent. The questionnaire responses were extracted from the app database in March 2020 for analysis. The patients’ demographic information, that is, age, gender, and education level, was extracted from the database of the web-based portal.

### Data Analysis

The research model was tested by partial least squares structural equation modelling (PLS-SEM) using the software program SmartPLS (version 3.0, SmartPLS GmbH) [39,45,46]. The PLS-SEM is commonly used to model the dynamic relationships between antecedent variables and dependent variables, thereby addressing the limitation of the multiple regression model with a relatively fixed relationship between variables and multicollinearity issues [47]. Moreover, the average number of latent variables in PLS-SEM is 7.94, which is much higher than 4.70 in covariance-based SEM [46]. Therefore, PLS-SEM is more conducive to solving more complex models. In addition, compared with the covariance-based SEM, PLS-SEM has a high level of statistical power even when the sample size is relatively small [48]. This is very practical for studies that have difficulties in recruiting research subjects, especially for those that are inherently complex and sensitive, like this study. The measurement model was tested by assessing reliability and validity [39,49]. For reflective constructs, the indicator reliability was assessed by the indicator loadings and collinearity statistics, that is, outer variance inflation factor (VIF) values. The construct reliability was assessed by Cronbach alpha and composite reliability. The convergent validity was assessed by the average variance extracted. The discriminant validity was assessed by the Fornell-Larcker criterion, cross-loading, and heterotrait-monotrait ratio [39,50]. For formative constructs, reliability was an irrelevant assessment criterion [49]. The indicator validity was assessed by the indicator weights and VIF. The discriminate validity was assessed by interconstruct correlations. The structural model was tested by path coefficients (β), variance explained (R^2^), effect size (*f*^2^), and the blindfolding-based cross-validated redundancy measure (Q^2^). Path coefficients (β) measured the direct effect of a variable assumed to be a cause on another variable assumed to be an effect [51]—a positive β value referred to a positive association and vice versa. Variance explained (R^2^) referred to in-sample predictive power, which measured a model’s explanatory power [52,53]. Effect size (*f*^2^) explained the changes before and after an exogenous construct is included and excluded from the model [54]. The *f*^2^ values of 0.02, 0.15, and 0.35 were considered as small, medium, and large effects, respectively. The Q^2^ value was used to assess the PLS path model’s predictive accuracy [55,56].

## Results

### Survey Participants’ Characteristics

A total of 141 participants completed the questionnaire survey. Four of them gave the same answer to all the questions. Another 8 provided the same answer to questions 16 and 17, which were opposite to each other in nature. These responses were considered invalid responses and were excluded from further data analysis. Therefore, 129 responses were used in the statistical analysis. There are 2 requirements regarding the sample size. First, the ratio of the sample size to the number of parameters should be greater than 5:1 [57]; second, the sample size should be greater than 10 times the largest number of either the formative items used to measure a single construct or the largest number of paths the latent variable has in the model [45,47]. In this study, the number of parameters is 7, the number of formative items is 3, and the number of paths the latent variable has is 10. Therefore, our sample size of 129 responses surpassed the 2 threshold requirements. The participants’ ages ranged from 34 to 79 years, with a median of 53 years. Two times more males than females participated in the survey. Approximately 69.8% (90/129) of the respondents were in the age group of 40-59 years, 62.8% (81/129) were in the workforce, and 75.9% (98/129) had an education level of high school or above (Table 2).

**Table 2:** Demographics of the participants (N=129).

Characteristics			Values, n (%)
**Gender**			
	Male	98 (76.0)	
	Female	31 (24.0)	
**Age (years)**			
	<40	7 (5.4)	
	40-49	48 (37.2)	
	50-59	42 (32.6)	
	60-69	23 (17.8)	
	≥70	9 (7.0)	
**Employment status**			
	Employed	66 (51.2)	
	Self-employed	15 (11.6)	
	Unemployed	7 (5.4)	
	Retired	29 (22.5)	
	No response	12 (9.3)	
**Education level**			
	Primary and middle school	22 (17.1)	
	High school	25 (19.4)	
	University/college/graduate	69 (53.5)	
	Postgraduate	4 (3.1)	
	No response	9 (6.9)	

### Descriptive Statistics of the Constructs

The mean scores of all the latent variables were positive, that is, close to or over 4 in the 5-point Likert scale (Table 1). In particular, the first item to measure the continuous use intention, “I intend to continue using the Blood Pressure Assistant rather than discontinue its use” reached a high mean score of 4.519, suggesting that the participants had high intention to continue to use the mHealth service.

### Measurement Model Validation

For the reflective constructs, that is, perceived usefulness, user satisfaction, perceived health status, and continuous use intention, each item was loaded above the threshold value of 0.708 on its respective construct and was significant at *P*=.01 (Table 3 and Multimedia Appendix 1). All VIF values were less than 3, indicating some correlation but not enough to be overly concerned about [58]. These confirmed the indicator reliability. All Cronbach alpha and composite reliability values were above .700, which confirmed the construct reliability. All average variance extracted values were more than 0.500, with their square root presented on the diagonal (Table 4). The average variance extracted of each construct was greater than its squared correlations with other constructs, confirming the convergent validity. The cross-loadings of each indicator on other constructs were lower than that on its designated construct, and each indicator loaded highest on its own construct. All the heterotrait-monotrait values were below 0.900. These confirmed the discriminant validity of the constructs [59]. For the formative constructs, that is, information quality, system quality, and service quality, each item was weighted above the threshold value of 0.200 on its respective construct and was significant at *P*=.01. All the VIF values were below 3, confirming indicator validity [58]. The correlations between the formative and all the other constructs were less than 0.700, confirming discriminant validity.

**Table 3:** Descriptive statistics of the construct, internal reliability, and convergent validity.

Construct and scale, item code		Standardized loading/weight	Variance inflation factor (outer)	Cronbach alpha		Composite reliability		Average variance extracted	
**Information quality (IQ), Formative**					N/A^a^		N/A		0
	IQ1	0.292	1.632						
	IQ2	0.555	1.513						
	IQ3	0.395	1.284						
**System quality (SysQ), Formative**					N/A		N/A		0
	SysQ1	0.428	2.032						
	SysQ2	0.649	2.032						
**Service quality (SerQ), Formative**					N/A		N/A		0
	SerQ1	0.369	2.284						
	SerQ2	0.693	2.284						
**Perceived usefulness (PU), Reflective**					.829		0.921		0.854
	PU1	0.917	2.006						
	PU2	0.931	2.006						
**User satisfaction (US), Reflective**					.787		0.903		0.824
	US1	0.900	1.725						
	US2	0.915	1.725						
**Perceived health status (PHS), Reflective**					.771		0.867		0.684
	PHS1	0.828	1.759						
	PHS2	0.833	1.710						
	PHS3	0.821	1.415						
**Continuous use intention (CUI), Reflective**					.703		0.860		0.756
	CUI1	0.946	1.417						
	CUI2	0.786	1.417						

^a^N/A: not applicable.

**Table 4:** Discriminant validity.^a^

Constructs	Continuous use intention	Information quality	Perceived health status	Perceived usefulness	Service quality	System quality	User satisfaction
Continuous use intention	*0.870*	—^b^	—	—	—	—	—
Information quality	0.489	—	—	—	—	—	—
Perceived health status	0.466	0.430	*0.827*	—	—	—	—
Perceived usefulness	0.578	0.618	0.458	*0.924*	—	—	—
Service quality	0.390	0.525	0.321	0.496	—	—	—
System quality	0.452	0.572	0.293	0.699	0.367	—	—
User satisfaction	0.574	0.514	0.513	0.714	0.426	0.478	*0.908*

^a^The values presented in italics on the diagonal are the square roots of average variance extracted (the variance shared between the constructs and their measures). Off-diagonal values are the correlation coefficients for each construct in the relevant rows and columns.

^b^Not applicable.

### Structural Model and Hypothesis Testing

**Figure 2:**
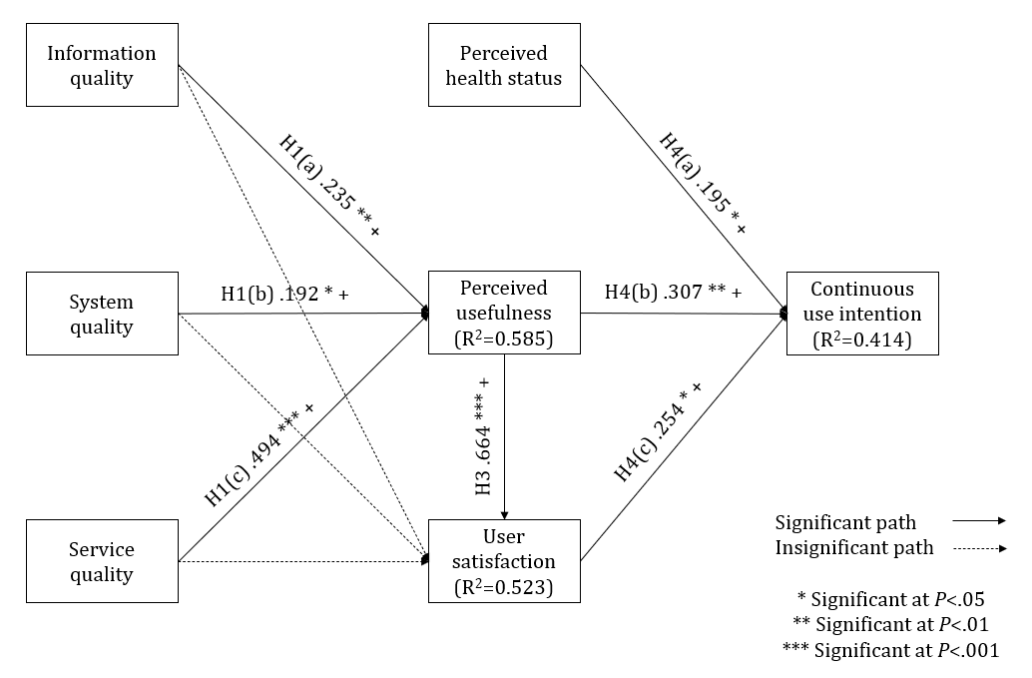
The validated theoretical model. "H" refers to the hypotheses in this study. +: positive association; –: negative association.

For the path coefficient (β) and variance explained (R^2^), our results showed that information quality, system quality, and service quality were all positively associated with perceived usefulness. Perceived usefulness and user satisfaction were positively associated with continuous use intention. Perceived usefulness was positively associated with user satisfaction. However, opposite to the original hypothesis, perceived health status was also positively associated with continuous use intention. Therefore, hypotheses 1(a), 1(b), 1(c), 3, 4(b), and 4(c) were confirmed but hypotheses 2(a), 2(b), 2(c), and 4(a) were not confirmed (Figure 2, Table 5, and Multimedia Appendix 2). The effects of service quality on perceived usefulness and the effects of perceived usefulness on user satisfaction were both large, while other paths showed small effects. The Q^2^ values for all the 3 endogenous constructs were positive, that is, perceived usefulness (0.400), user satisfaction (0.337), and continuous use intention (0.144), which confirmed the model’s predictive accuracy.

**Table 5:** Structural model validation and hypothesis testing results.

Path	Standard error	*t* *(df)*	*P* value	Effect size (*f*^2^)	Hypothesis testing
H1(a): IQ→PU^a^	0.083	2.839 (9)	.005	Small (0.074)	Confirmed
H1(b): SysQ→PU^b^	0.084	2.293 (9)	.02	Small (0.064)	Confirmed
H1(c): SerQ→PU^c^	0.089	5.525 (9)	<.001	Large (0.391)	Confirmed
H2(a): IQ→US^d^	0.094	1.193 (9)	.23	None (0.014)	Not confirmed
H2(b): SysQ→US^e^	0.085	0.761 (9)	.45	None (0.006)	Not confirmed
H2(c): SerQ→US^f^	0.087	0.852 (9)	.39	None (0.005)	Not confirmed
H3: PU→US^g^	0.100	6.632 (9)	<.001	Large (0.384)	Confirmed
H4(a): PHS→CUI^h^	0.092	2.121 (9)	.03	Small (0.047)	Not confirmed
H4(b): PU→CUI^i^	0.107	2.879 (9)	.004	Small (0.077)	Confirmed
H4(c): US→CUI^j^	0.122	2.082 (9)	.04	Small (0.049)	Confirmed

^a^Hypothesis 1(a): Information quality is positively associated with patients’ perceived usefulness of mobile health services.

^b^Hypothesis 1(b): System quality is positively associated with patients’ perceived usefulness of mobile health services.

^c^Hypothesis 1(c): Service quality is positively associated with patients’ perceived usefulness of mobile health services.

^d^Hypothesis 2(a): Information quality is positively associated with user satisfaction of mobile health services.

^e^Hypothesis 2(b): System quality is positively associated with user satisfaction of mobile health services.

^f^Hypothesis 2(c): Service quality is positively associated with user satisfaction of mobile health services.

^g^Hypothesis 3: Perceived usefulness of mobile health services is positively associated with user satisfaction with such services.

^h^Hypothesis 4(a): Perceived health status is negatively associated with continuous use intention of mobile health services.

^i^Hypothesis 4(b): Perceived usefulness is positively associated with continuous use intention of mobile health services.

^j^Hypothesis 4(c): User satisfaction is positively associated with continuous use intention of mobile health services.

## Discussion

### Principal Results

This empirical study proposes a theoretical model to predict and explain patients’ intention to continue using mHealth services for self-management of chronic conditions. This model is derived from a hybrid model synthesized from the IS continuance model and IS success model [27,28]. The statistical assessment confirmed the reliability and validity of its measurement scale (Table 3 and Table 4). Six out of 10 original hypotheses about the relationships among 7 variables were confirmed (Table 5). Information quality, system quality, and service quality have a significant positive influence on perceived usefulness but not on user satisfaction. Perceived usefulness has a significant positive influence on user satisfaction. Perceived usefulness and user satisfaction have positive effects on participants’ intention to continue using the mHealth services.

Contrary to the original hypothesis of a negative association, the patients’ perceived health status is positively associated with their continuous use intention. This may be explained by the motivation effect of positive reward stemming from decreased blood pressure, which can directly motivate patients to form a virtuous cycle in self-management of chronic conditions, that is, the better the result in blood pressure control, the more actively a patient will use the app. Another possible reason is that frequent use of self-monitoring mechanisms, that is, entering and monitoring their own health data, has become a daily routine or habit. Thus, it is no longer influenced by intention, which only impacts voluntary use [60]. Lee et al [25] found that after introducing the self-monitoring function, the negative slope of the downward trend in mHealth service usage was alleviated in patients for self-management of their general health, which supported the long-term positive effects of self-monitoring for chronic conditions. However, according to the health belief model [61], which defines the key factors that influence health behaviors, one of the most important factors is the perceived severity, that is, belief of the consequence of the conditions. Therefore, once a patient’s blood pressure is controlled, the person’s perceived health status is improved and the perceived severity is relieved; therefore, the use intention will decrease. We consider that this paradox may be moderated by health literacy [15,62]. For patients with high health literacy, awareness of the negative consequences of not using the app coupled with the motivating effect brought by the perceived usefulness will motivate them to use the app continuously. Conversely, patients with low health literacy, owing to their lack of understanding of the negative effect of discontinued use, may not intend to use the app after a period or when their blood pressure is controlled. This is similar to the findings of Guo et al [23] that a patient’s health consciousness has a significant positive impact on the relationship between social media influence and continuous use intention. Therefore, health literacy could be a moderator for the relationship between perceived health status and continued use intention, which can be further tested in a follow-up study.

The results also support the importance of providing feedback to patients about their health status that is reflected by the vital signs they uploaded in mHealth services [8]. Awareness of their own health status, particularly if the disease is deteriorating, will drive them to formulate an intention to continuously use the mHealth services. Conversely, when the health status is improving, patients might feel bored by repeatedly entering and uploading data day after day through the mHealth app, as reported by Biduski et al [63]. Without the feedback and awareness about their own health status, patients would assume they are in good health and gradually lose the intention to use the services.

### Contributions of This Study

#### Theoretical Contribution

Our paper, for the first time, integrated 2 classic information system models for investigating the continuous use of mHealth services to support patient self-management of chronic conditions in the hypertension context [64]. The IS continuance model emphasizes the impact of expectation on perceived usefulness and user satisfaction based on the expectation-confirmation theory [27,29]. However, it does not clearly indicate which factors of the expectations are confirmed. The IS success model clarifies and supplements the 3 factors of the expectation: information quality, system quality, and service quality [28].

As hypothesized, our study confirms the cascading effects of 3 antecedent variables, that is, information quality, system quality, and service quality, on a patient’s continuous use intention through the intermittent variables, that is, perceived usefulness and user satisfaction. Our findings are in line with Wu’s findings that information quality and service quality have a significant positive impact on perceived usefulness [31]. In our study, service quality plays a more important role than information quality and system quality, which may indicate the importance of ongoing support from health care providers for patients to continuously use the mHealth app. A previous study in the same population showed that the relationship with the health care provider is one of the determinants for the patient’s intention to use the same app [13]. Biduski et al [63] also found that the most satisfactory experience using mHealth services in self-management of chronic conditions mainly occurred in the first week and concentrated on the practicality of treatment monitoring. The service in Wu’s study [31] was a web-based health community. Patients usually communicate with their doctors and seek relevant information through a web-based community. Our service provides patients with a disease-focused intervention specifically for the management of hypertension. In addition to obtaining information, the patients also require ongoing support provided by a health manager, which is an important component of service. This is also reflected in the positive evaluation of patients in questions 6 and 7, that is, “The health manager can resolve problems which I encountered when using the Blood Pressure Assistant” (4.3/5) and “The health manager gives me individual guidance in the follow-up phone calls” (4.1/5). The services provided by the health manager include guidance and assurance on self-management when abnormal blood pressure levels are detected. The health manager would call the patients or their family to discuss and modify the management plan. These interactions give patients the comfort that they receive full attention and that they receive high-quality service from their health care providers. These interactions enhance their rapport with health care providers and trust and recognition of service quality so that patients can perceive the usefulness of mHealth and generate the intention to make continuous use of the app. Our finding confirms the vital role that health care providers play in ensuring that patients use the mHealth service continuously.

The finding that the system quality has less influence on perceived usefulness in comparison with information and service quality may be explained by the complexity and persistent effort required for the management of chronic conditions [65,66]. Unlike using other systems, patients are more concerned about whether the service and information provided by the app can help them control the disease. This may also explain why user satisfaction is not directly affected by information quality, service quality, and system quality but indirectly through perceived usefulness.

#### Practical Contribution

Although the rapid development of mHealth services and the initial acceptance by patients have brought new opportunities for managing chronic conditions, owing to the lasting nature of these conditions and the long-term requirements for behavior modification, only patients who continue to use the mHealth service can benefit from such services. Our research results show that 3 independent factors, namely, information quality, system quality, and service quality determine patients’ intention to continue to use mHealth services through the mediation of perceived usefulness and user satisfaction. In the context of this study, service quality (the assistance and feedback of health care providers), information quality (the provision of reliable and relevant information), and system quality (an easy-to-learn and easy-to-operate system) are essential for patients’ continuous use of an mHealth service. Therefore, to successfully introduce mHealth innovation into self-management of chronic conditions, it is necessary to focus on improving the responsibility and ability of health care providers and to continue to provide and update the educational information for patients to improve their awareness of the disease threat and effective self-management methods. These strategies will further enable patients to understand the usefulness of mHealth services and manage their chronic conditions by using the services continuously.

### Limitations and Future Work

The first limitation of this study is the limited geographical and social coverage of the study population, which is limited to an underdeveloped area in China. The sample size is relatively small, with only 129 patients, despite reaching the threshold for theoretical sampling. The mHealth app is purposely built to test the feasibility of hypertension control by using an app; thus, its usability may not be representative of all mHealth apps for any type of chronic conditions. Thus, those implementing the findings should be cautious about generalizing these findings. Second, the latent variables were selected based on the group’s previous research experience and literature review; other factors may also need to be captured in the model that we tested, for example, health literacy. Third, as a trade-off for avoiding survey fatigue and improving response rates [28], many constructs were measured by 2 items instead of 3 items suggested by Hair et al [39], which were suboptimal from the perspective of measurement theory. Since the questionnaire was answered voluntarily by the users of the mHealth service, the results cannot avoid the positive bias in sampling, that is, nonusers would not answer the questionnaires. In addition, no control or moderating variables, that is, patient characteristics, were included in the structural model for the sake of meeting the threshold number of measures in SEM. Future research can investigate the impact of the control or moderating variables, that is, perceived risk and patient-doctor relationship [13,15] on perceived usefulness, user satisfaction, or continuous use intention. Future research needs to also be conducted as an empirical field study on the long-term effects of mHealth services in natural settings to derive generalizable insight for improving the practice of implementing mHealth services.

### Conclusions

This study developed a model and questionnaire survey instrument to measure the success of patients’ continuous use of mHealth services for self-management of chronic conditions. This study shows that patients’ intention to continue to use an mHealth service for self-management of chronic conditions is influenced by information quality, system quality, and service quality, through the mediation of perceived usefulness and user satisfaction. The patients’ perceived health status also has a significant positive influence on their continuous use intention. The validated model and measurement scale are useful for the routine evaluation of patients’ continuous use of mHealth services, which is also important for evaluating the operational effect of the mHealth program. The research model and the questionnaire survey instrument developed can be used for routine identification of the areas of mHealth management provided to the patients that support their use of the mHealth services that need improvement, that is, information quality, mobile app usability, and technical and health care provider services. These findings also enrich the body of knowledge of continuous use of mHealth for self-management of chronic conditions. Future research can apply the model and questionnaire survey instrument to other types of mHealth services.

## Appendix

Multimedia Appendix 1 Cross-loading of the latent variables.

Multimedia Appendix 2 The direct, indirect, and total effects of antecedent and dependent variables on the other dependent variables.

## Abbreviations

IS: information systems
mHealth: mobile health
PLS-SEM: partial least squares structural equation modelling
VIF: variance inflation factor
