# Supplementary material for: Measuring Success of Patients’ Continuous Use of Mobile Health Services for Self-management of Chronic Conditions: Model Development and Validation
Source: J Med Internet Res. 2021 Jul 13;23(7):e26670. doi: 10.2196/26670 (PMC8317034; doi:10.2196/26670)
Supplement: Multimedia Appendix 1 [file jmir_v23i7e26670_app1.pdf]

Multimedia Appendix 1

Cross-loading of the latent variables.

	CUI	IQ	PHS	PU	SerQ	SysQ	US
**CUI1**	**0.946**	0.521	0.45	0.635	0.485	0.45	0.623
**CUI2**	**0.786**	0.275	0.349	0.287	0.246	0.153	0.299
**IQ1**	0.392	**0.784**	0.236	0.474	0.392	0.474	0.415
**IQ2**	0.436	**0.868**	0.413	0.512	0.406	0.422	0.475
**IQ3**	0.336	**0.732**	0.333	0.494	0.588	0.386	0.327
**PHS1**	0.339	0.257	**0.828**	0.333	0.198	0.208	0.422
**PHS2**	0.377	0.349	**0.833**	0.424	0.273	0.275	0.396
**PHS3**	0.43	0.438	**0.821**	0.375	0.25	0.303	0.452
**PUU1**	0.556	0.555	0.413	**0.917**	0.643	0.433	0.576
**PUU2**	0.514	0.587	0.433	**0.931**	0.648	0.482	0.737
**SerQ1**	0.458	0.549	0.269	0.622	**0.889**	0.392	0.422
**SerQ2**	0.408	0.534	0.28	0.676	**0.97**	0.321	0.464
**SysQ1**	0.37	0.472	0.316	0.426	0.312	**0.89**	0.398
**SysQ2**	0.357	0.498	0.287	0.484	0.36	**0.954**	0.393
**US1**	0.522	0.455	0.51	0.605	0.421	0.356	**0.9**
**US2**	0.52	0.478	0.425	0.687	0.446	0.415	**0.915**

IQ: Information quality; SysQ: System quality; SerQ: Service quality; PU: Perceived usefulness; US: User satisfaction; PHS: Perceived health status; CUI: Continuous use intention
