# Supplementary material for: Measuring Success of Patients’ Continuous Use of Mobile Health Services for Self-management of Chronic Conditions: Model Development and Validation
Source: J Med Internet Res. 2021 Jul 13;23(7):e26670. doi: 10.2196/26670 (PMC8317034; doi:10.2196/26670)
Supplement: Multimedia Appendix 2 [file jmir_v23i7e26670_app2.pdf]

Multimedia Appendix 2

The direct, indirect and total effects of antecedent and dependent variables on the other dependent variables.

Relationships (A predicts B)		Direct effects	Indirect effects	Total effects
A	B			
Information quality	Perceived usefulness	0.235	0.000	0.235
System quality	Perceived usefulness	0.192	0.000	0.192
Service quality	Perceived usefulness	0.494	0.000	0.494
Information quality	User satisfaction	0.112	0.156	0.268
System quality	User satisfaction	0.064	0.127	0.191
Service quality	User satisfaction	-0.074	0.328	0.254
Perceived usefulness	User satisfaction	0.664	0.000	0.664
Information quality	Continuous use intention	0.000	0.140	0.140
System quality	Continuous use intention	0.000	0.108	0.108
Service quality	Continuous use intention	0.000	0.216	0.216
Perceived health status	Continuous use intention	0.195	0.000	0.195
Perceived usefulness	Continuous use intention	0.254	0.000	0.254
User satisfaction	Continuous use intention	0.307	0.169	0.476
